# Left Ventricular Systolic Dysfunction in NBCe1-B/C-Knockout Mice

**DOI:** 10.3390/ijms25179610

**Published:** 2024-09-05

**Authors:** Clayton T. Brady, Aniko Marshall, Lisa A. Eagler, Thomas M. Pon, Michael E. Duffey, Brian R. Weil, Jennifer K. Lang, Mark D. Parker

**Affiliations:** 1 Department of Physiology and Biophysics, Jacobs School of Medicine and Biomedical Sciences, State University of New York: The University at Buffalo, Buffalo, NY 14203, USAbweil@buffalo.edu (B.R.W.); 2 Division of Cardiovascular Medicine and the Clinical and Translational Research Center, State University of New York: University at Buffalo, Buffalo, NY 14203, USA; lavathy@buffalo.edu (L.A.E.); tpon@buffalo.edu (T.M.P.); jklang@buffalo.edu (J.K.L.); 3 Veterans Affairs Western New York Health Care System, Buffalo, NY 14215, USA; 4 Department of Biomedical Engineering, State University of New York: University at Buffalo, Buffalo, NY 14260, USA; 5 Department of Pharmacology and Toxicology, State University of New York: University at Buffalo, Buffalo, NY 14203, USA; 6 Department of Medicine, State University of New York: University at Buffalo, Buffalo, NY 14203, USA; 7 Department of Ophthalmology, Jacobs School of Medicine and Biomedical Sciences, State University of New York: The University at Buffalo, Buffalo, NY 14209, USA

**Keywords:** NBCe1, acid–base, pRTA, heart failure, contractility, calcium

## Abstract

Congenital proximal renal tubular acidosis (pRTA) is a rare systemic disease caused by mutations in the *SLC4A4* gene that encodes the electrogenic sodium bicarbonate cotransporter, NBCe1. The major NBCe1 protein variants are designated NBCe1-A, NBCe1-B, and NBCe1-C. NBCe1-A expression is kidney-specific, NBCe1-B is broadly expressed and is the only NBCe1 variant expressed in the heart, and NBCe1-C is a splice variant of NBCe1-B that is expressed in the brain. No cardiac manifestations have been reported from patients with pRTA, but studies in adult rats with virally induced reduction in cardiac NBCe1-B expression indicate that NBCe1-B loss leads to cardiac hypertrophy and prolonged QT intervals in rodents. NBCe1-null mice die shortly after weaning, so the consequence of congenital, global NBCe1 loss on the heart is unknown. To circumvent this issue, we characterized the cardiac function of NBCe1-B/C-null (KO_b/c_) mice that survive up to 2 months of age and which, due to the uninterrupted expression of NBCe1-A, do not exhibit the confounding acidemia of the globally null mice. In contrast to the viral knockdown model, cardiac hypertrophy was not present in KO_b/c_ mice as assessed by heart-weight-to-body-weight ratios and cardiomyocyte cross-sectional area. However, echocardiographic analysis revealed reduced left ventricular ejection fraction, and intraventricular pressure–volume measurements demonstrated reduced load-independent contractility. We also observed increased QT length variation in KO_b/c_ mice. Finally, using the calcium indicator Fura-2 AM, we observed a significant reduction in the amplitude of Ca^2+^ transients in paced KO_b/c_ cardiomyocytes. These data indicate that congenital, global absence of NBCe1-B/C leads to impaired cardiac contractility and increased QT length variation in juvenile mice. It remains to be determined whether the cardiac phenotype in KO_b/c_ mice is influenced by the absence of NBCe1-B/C from neuronal and endocrine tissues.

## 1. Introduction

**Figure 1:**
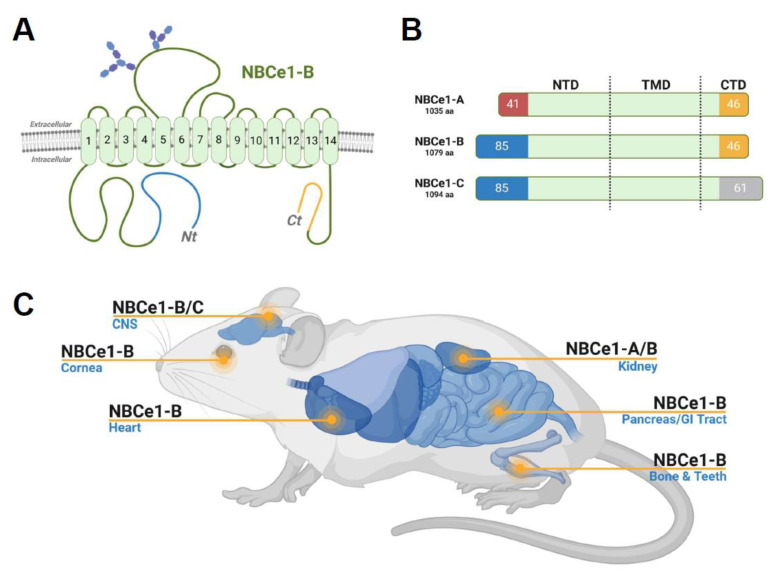
Structure and expression of NBCe1 major isoforms. (**A**) An illustration of NBCe1-B protein topology. All NBCe1 isoforms have 14 transmembrane spans (TM1–14), with soluble N-terminal and C-terminal (Nt and Ct) domains located within the cytoplasm. A glycosylated extracellular loop joins TMs 5 and 6. (**B**) An illustration of sequence differences between NBCe1 isoforms. Due to an alternative upstream promoter that controls NBCe1-B translation, there is a different 85-amino acid (aa) Nt sequence in NBCe1-B (shown in blue) that replaces the first 41 aa residues of NBCe1-A (shown in red). NBCe1-C is identical to NBCe1-B except that the last 46 aa residues of the Ct sequence in NBCe1-B (shown in yellow) are replaced by a different 61 aa sequence (shown in grey) as a consequence of alternative splicing. (**C**) An illustration of the expression pattern of NBCe1 protein isoforms. The figure was created using BioRender.com.

Congenital proximal renal tubular acidosis (pRTA) is a rare, systemic disease characterized by severe acidemia resulting from impaired proximal tubule bicarbonate (HCO_3_^−^) transport. In addition to low plasma pH and [HCO_3_^−^], the 20 pRTA case reports to date describe several other disease features such as growth retardation, ocular pathologies (band keratopathy, cataracts, and glaucoma), dental abnormalities, intellectual impairment, bilateral basal ganglia calcification, epilepsy, migraines, and muscle weakness [1,2,3,4,5,6,7,8,9,10,11,12,13,14,15,16,17,18,19,20]. In these reports, various mutations in the *SLC4A4* gene, which encodes the electrogenic sodium/bicarbonate (Na^+^/2HCO_3_^−^) cotransporter (NBCe1), were determined to cause congenital pRTA. Notably, *SLC4A4* expresses three major variants of NBCe1 designated A–C (Figure 1: reviewed in [21]). NBCe1-A is only expressed in the kidney proximal tubule, and it is the loss of NBCe1-A that is largely responsible for the severe urinary HCO_3_^−^ wasting and acidemia characteristic of congenital pRTA [22,23]. NBCe1-B, while being a minor renal variant, is the major extrarenal variant with expression in cells of the pancreas, intestine, bone, brain, and heart [23,24,25]. Finally, NBCe1-C expression has only been reported in the brains of rats [26], whereas no NBCe1-C transcripts or protein expression has been detected in human tissue [27], and its relevance is currently unknown.

The identification of *SLC4A4* mutations as the cause of congenital pRTA has prompted the development of several mouse models that have advanced our understanding of the underlying pathologies associated with congenital pRTA and highlighted the importance of NBCe1. Among these include the total-NBCe1-knockout mouse (KO_total_) [28], the NBCe1-A-specific-knockout mouse (KO_a_) [22], and the NBCe1-B/C-knockout mouse (KO_b/c_) [29]. There are two important findings to note from the study of these mouse models. Firstly, both KO_total_ and KO_a_ mice develop spontaneous, severe metabolic acidosis due to loss of the HCO_3_^−^ transport activity of NBCe1-A in the kidney proximal tubule. Secondly, since KO_b/c_ mice maintain kidney NBCe1-A expression, they do not develop metabolic acidosis. However, both KO_total_ and KO_b/c_ mice have similar phenotypic features (except for metabolic acidosis) including developmental defects, ocular abnormalities, enamel hypomineralization, and impacted intestines [28,29]. Moreover, both KO_total_ and KO_b/c_ mice have increased mortality (50% mortality at 12 and 35 days, respectively), whereas KO_a_ mice live into adulthood [22,28,29]. The conclusions drawn from these findings is that the organ-specific loss of NBCe1-B/C is responsible for the extrarenal phenotypic features of congenital pRTA (as opposed to the overt acidemia), and furthermore, it is the loss of NBCe1-B/C that is the major contributor to the increased mortality seen in total NBCe1 deletion. Nevertheless, the contribution of NBCe1 to the physiology of extrarenal organ systems remains understudied.

Of increasing interest is the role of NBCe1-B in cardiac physiology. In cardiomyocytes, NBCe1-B imports base into the sarcoplasm, working in conjunction with the electroneutral NBC (NBCn1) and the Na^+^/H^+^ exchanger (NHE1), to neutralize intracellular acids and maintain intracellular pH balance (reviewed in [30]). However, compared to the 1Na^+^:1(H^+^ or HCO_3_^−^) transport stoichiometry of NBCn1 and NHE1, NBCe1-B imports the equivalent of two HCO_3_^−^ ions (actually one CO_3_^=^ ion [31]) per single Na^+^ ion. Thus, NBCe1 imports half as many Na^+^ ions per base equivalent compared to NBCn1 or NHE1. This difference in stoichiometry has led to the description of NBCe1-B as “Na^+^ sparing” [30]. Consequently, a reduction in NBCe1-B expression/activity, with compensatory upregulation of NHE1 and/or NBCn1 activity [32,33], is hypothesized to cause an increased intracellular Na^+^ load that impairs the Ca^2+^-extrusion activity of the Na^+^/Ca^2+^ exchanger (NCX1), resulting in Ca^2+^-mediated hypertrophic growth, electrophysiologic disruption, and/or functional changes [30]. Indeed, reduction in cardiac NBCe1-B expression via virally induced inhibition in adult rats led to cardiac hypertrophy [32]. In addition, reduced cardiac NBCe1-B expression resulted in increased QT interval duration, which correlated with prolongation of the action potential duration (APD) in isolated rat cardiomyocytes [32]. Finally, in the spontaneously hypertensive rat model of pre-existing cardiac hypertrophy, NBCe1-B activity was reduced, whereas NBCn1 activity was upregulated; an effect found to be mediated by angiotensin II signaling [33], overall supporting the hypothesis that the loss of NBCe1-B leads to cardiac dysfunction.

However, despite the growing evidence supporting the importance NBCe1-B in cardiac physiology, no cardiac manifestations have been described in case reports of congenital pRTA patients, and no phenotypes have been linked to individuals with homozygous NBCe1-B/C-specific nucleotide changes [1,2,3,4,5,6,7,8,9,10,11,12,13,14,15,16,17,18,19,20]. The extent to which this may be explained by a high perinatal mortality has never been addressed. Thus, the effect of congenital absence of cardiac NBCe1-B remains unclear. While congenital pRTA is an exceedingly rare disease, heart failure with reduced ejection fraction (i.e., systolic dysfunction) is a significant cause of morbidity and mortality in the United States with an estimated >3 million adults effected [34], underscoring the need for novel therapeutics to abate the development and progression of heart failure. Therefore, understanding the underlying molecular mechanisms of systolic dysfunction is a valuable target.

Accordingly, the major aim of this study is to characterize the cardiac function of the KO_b/c_ mouse, which is uniquely suited for investigation of the effect of congenital, global NBCe1-B loss without the confounding effect of metabolic acidosis seen in KO_total_ mice. To this end, we assessed the cardiac function of 4–5-week-old wild-type (WT) and KO_b/c_ mice using echocardiography, intraventricular admittance catheter-derived pressure–volume analysis, and electrocardiography (ECG). We additionally performed histological examination of hearts for evidence of hypertrophy. Finally, we assessed the effect of NBCe1-B absence on [Ca^2+^]_i_ dynamics using quantitative high-speed fluorescent recordings of paced Fura-2 AM-loaded cardiomyocytes isolated from WT and KO_b/c_ hearts.

## 2. Results

### 2.1. Reduced Ejection Fraction with Diminished Systolic Function in KO_b/c_ Mice

**Figure 2:**
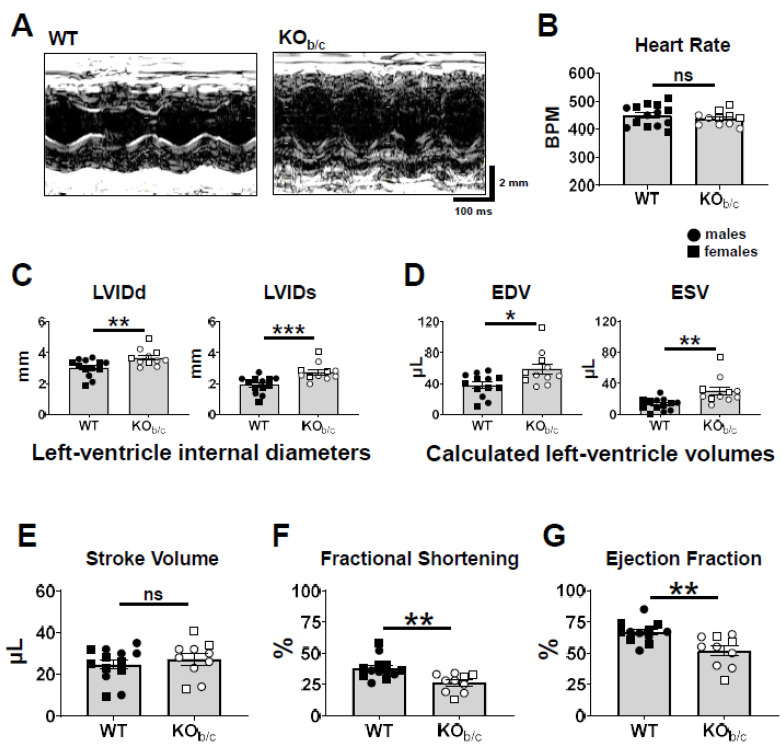
Echocardiography demonstrates impaired left ventricular function in KO_b/c_ mice. (**A**) Representative cross-sectional M-mode images of the left ventricle of WT and KO_b/c_ mice between 4–5 weeks of age. (**B**) Heart rates were titrated to between ~400–500 BPM via isoflurane anesthesia. (**C**) KO_b/c_ mice were found to have significantly greater left ventricle internal diameters during diastole (LVIDd) and systole (LVIDs). (**D**) KO_b/c_ mice also had significantly greater end-diastolic volume (EDV) and end-systolic volume (ESV) than WT mice as calculated from LVID measurements. (**E**) There was no significant difference in stroke volume between WT and KO_b/c_ mice. (**F**) The fractional shortening of KO_b/c_ mice was significantly less than that of WT mice. (**G**) The ejection fraction of KO_b/c_ mice was significantly less than that of WT mice. Data presented as mean ± SEM, n = 11–14 per group. Outliers were defined a priori as any point >2 standard deviations from the mean and were excluded from analysis. WT outliers (*n*) were excluded from LVIDd (1), LVIDs (1), EDV (1), ESV (1), SV (1), FS (1), and EF (2) data sets. KO_b/c_ outliers (*n*) were excluded from heart rate (1), LVIDd (1), LVIDs (1), EDV (1), ESV (1), SV (2), FS (2), and EF (2) data sets. A significant difference between WT and KO_b/c_ groups is indicated in the figure by * *p* < 0.05, ** *p* < 0.01, and *** *p* < 0.001 calculated using Student’s unpaired 2-tailed *T*-test; ns (non-significant).

Echocardiography was used to assess cardiac function in WT and KO_b/c_ mice while under isoflurane anesthesia, with representative M-mode images obtained in WT and KO_b/c_ mice shown in Figure 2A. The heart rate of each mouse was maintained between ~400–500 BPM via titration of isoflurane (WT: 449 ± 10 BPM, KO_b/c_: 438 ± 7 BPM, *p* = 0.450, n = 14 and 11, respectively). The left ventricle internal diameters during diastole and systole (LVIDd and LVIDs) were both significantly greater in KO_b/c_ mice (LVIDd–WT: 3.0 ± 0.1 mm, KO_b/c_: 3.7 ± 0.2. mm, *p* = 0.010, n = 13 and 11, respectively; LVIDs–WT: 1.9 ± 0.1 mm, KO_b/c_: 2.8 ± 0.2 mm, *p* = 0.001, n = 13 and 11; Figure 2C). Using the LVIDd and LVIDs to calculate the end-diastolic volume (EDV) and the end-systolic volume (ESV), we further determined that the EDV and ESV were significantly greater in KO_b/c_ mice (EDV–WT: 38 ± 4 µL, KO_b/c_: 59 ± 6 µL, *p* = 0.011, n = 13 and 11, respectively; ESV–WT: 13 ± 2 µL, KO_b/c_: 30 ± 5 µL, *p* = 0.003, n = 13 and 11, respectively; Figure 2D). However, there was no significant difference in stroke volume between WT and KO_b/c_ mice (WT: 25 ± 2 µL, KO_b/c_: 27 ± 3 µL, *p* = 0.442, n = 13 and 10, respectively; Figure 2E). Finally, the fractional shortening (FS) and the ejection fraction (EF) were significantly reduced in KO_b/c_ mice (FS–WT: 38 ± 2%, KO_b/c_: 26 ± 2%, *p* = 0.001, n = 13 and 10, respectively; EF–WT: 67 ± 2%, KO_b/c_: 52 ± 4%, *p* = 0.003, n = 12 and 10, respectively; Figure 2F,G).

**Figure 3:**
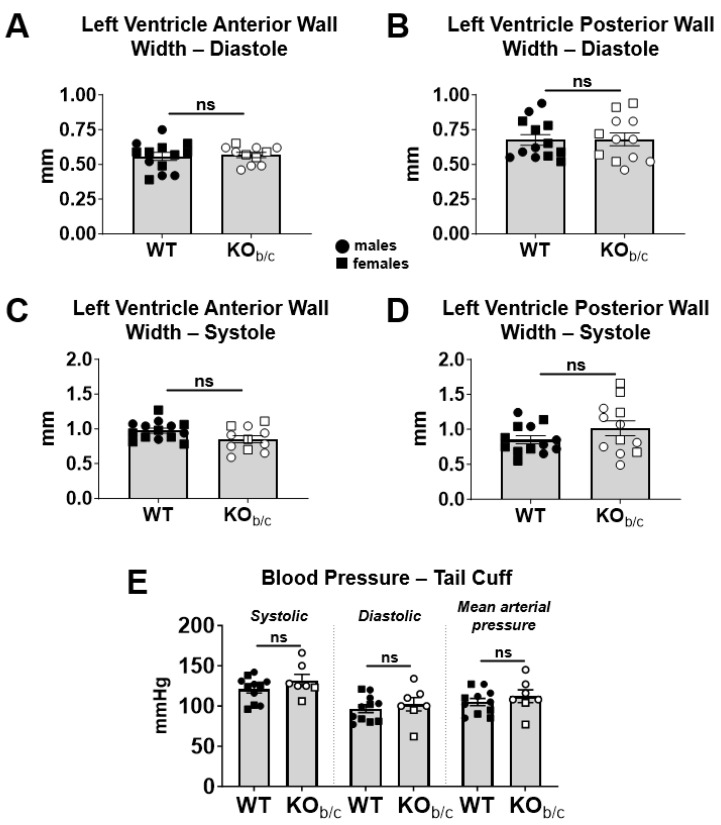
Impaired left ventricular function in KO_b/c_ mice is not attributable to differences in left ventricle wall thickness or systemic vascular resistance. During diastole there was no significant difference between the width of the WT and KO_b/c_ left ventricle anterior (**A**) or posterior (**B**) wall. Similarly, during systole, there was no significant difference between the width of the WT and KO_b/c_ left ventricle anterior (**C**) or posterior (**D**) wall. (**E**) There was no significant difference in systolic, diastolic, or mean arterial pressures of awake WT and KO_b/c_. Data presented as mean ± SEM, n = 11–14 per group (panels **A**–**D**) or 7–11 per group (panel **E**). Outliers were defined a priori as any point >2 standard deviations from the mean and were excluded from analysis. WT outliers (*n*) were excluded from LVAWd (1), LVPWd (1), and LVPWs (1) data sets. KO_b/c_ outliers (*n*) were excluded from LVAWd (1) and LVAWs (1) data sets. Statistical significance calculated using Student’s unpaired 2-tailed *T*-test; ns (non-significant).

To assess for possible structural abnormalities between WT and KO_b/c_ mice, we measured the width of the left ventricular anterior and posterior walls during diastole and during systole using M-mode echocardiography (Figure 3A–D). During diastole, there was no significant difference between the width of the left ventricular anterior or posterior walls (anterior wall width diastole—WT: 0.56 ± 0.03 mm, KO_b/c_: 0.57 ± 0.02 mm, *p* = 0.770, n = 13 and 11, respectively; posterior wall width diastole—WT: 0.68 ± 0.04 mm, KO_b/c_: 0.68 ± 0.04 mm, *p* = 0.935, n = 13 and 12, respectively; Figure 3A,B). Similarly, during systole, there was no significant difference between the width of the left ventricular anterior or posterior walls (anterior wall width systole—WT: 0.98 ± 0.03 mm, KO_b/c_: 0.85 ± 0.05 mm, *p* = 0.053, n = 14 and 11, respectively; posterior wall width systole—WT: 0.85 ± 0.06 mm, KO_b/c_: 1.02 ± 0.10 mm, *p* = 0.173, n = 13 and 12, respectively; Figure 3C,D). Additionally, we compared the blood pressure between WT and KO_b/c_ mice (Figure 3E) and found no significant difference between systolic pressure (WT: 121 ± 5 mmHg, KO_b/c_: 132 ± 7 mmHg, *p* = 0.214, n = 11 and 7, respectively), diastolic pressure (WT: 97 ± 5 mmHg, KO_b/c_: 102 ± 8 mmHg, *p* = 0.543, n = 11 and 7, respectively), or mean arterial pressure (WT: 105 ± 4 mmHg, KO_b/c_: 112 ± 7 mmHg, *p* = 0.401, n = 11 and 7, respectively). Together, these data suggest that the reduced ejection fraction observed in KO_b/c_ mice is not attributable to structural changes or increased systemic vascular resistance.

**Figure 4:**
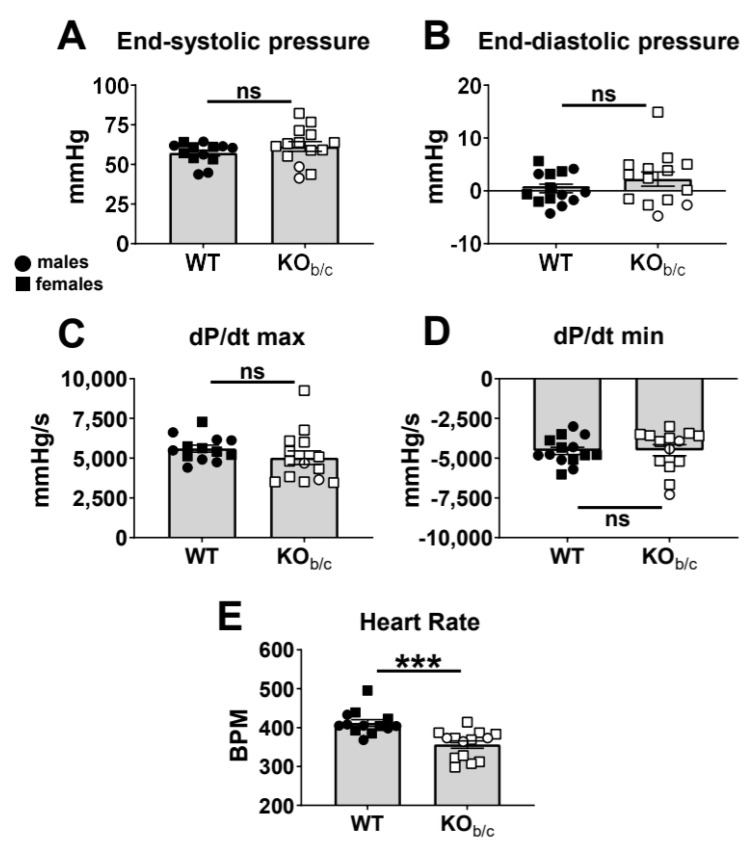
Left intraventricular pressure–volume (PV) assessment reveals no significant difference between WT and KO_b/c_ mice in load-dependent measures of contractility or relaxation. There was no significant difference between WT and KO_b/c_ left ventricular end-systolic pressure (**A**) or end-diastolic pressure (**B**). There was no significant difference between WT and KO_b/c_ mice in their left ventricular maximum rate of pressure change (dP/dt max, representing load-dependent contractility) (**C**) or in their minimum rate of pressure change (dP/dt min, representing load-dependent relaxation) (**D**). Heart rates were titrated to between ~300–500 BPM via isoflurane anesthesia (**E**). Data presented as mean ± SEM, n = 13–14 per group. Outliers were defined a priori as any point >2 standard deviations from the mean and were excluded from analysis. WT outliers (*n*) were excluded from end-systolic pressure (1), dP/dt max (1), and heart rate (1) data sets. KO_b/c_ outliers (*n*) were excluded from end-systolic pressure (1), end-diastolic pressure (1), dP/dt max (1), dP/dt min (1), and heart rate (1) data sets. A significant difference between WT and KO_b/c_ groups is indicated in the figure by *** *p* < 0.001 calculated using Student’s unpaired 2-tailed *T*-test; ns (non-significant).

Next, we assessed systolic and diastolic function of WT and KO_b/c_ mice by obtaining left intraventricular pressure–volume (PV) measurements. Figure 4 presents the results of baseline PV measurements (i.e., load-dependent parameters). We found no significant difference between baseline left ventricular end-systolic or end-diastolic pressures of WT and KO_b/c_ mice (baseline end-systolic pressure—WT: 51 ± 7 mmHg, KO_b/c_: 61 ± 3 mmHg, *p* = 0.277, n = 13 and 14, respectively; baseline end-diastolic pressure—WT: 0 ± 1 mmHg, KO_b/c_: 2 ± 1 mmHg, *p* = 0.264, n = 14 for both groups; Figure 4A,B). Similarly, we found no significant difference in the dP/dt maximum or minimum (load-dependent measures of systole and diastole, respectively) between WT and KO_b/c_ mice (dP/dt maximum—WT: 5604 ± 220 mmHg/s, KO_b/c_: 5030 ± 434 mmHg/s, *p* = 0.260, n = 13 and 14, respectively; dP/dt minimum—WT: −4546 ± 228 mmHg/s, KO_b/c_: −4503 ± 346 mmHg/s, *p* = 0.918, n = 14 for both groups; Figure 4C,D).

However, because of the invasive nature and depth of anesthesia required during this experiment (necessitating the lack of pedal reflex in individual mice), KO_b/c_ mice had a significantly lower heart rate than WT mice (WT: 412 ± 9 BPM, KO_b/c_: 356 ± 10 BPM, *p* < 0.001, n = 13 and 14, respectively). Since a slower heart rate leads to greater diastolic filling (preload) and can impact load-dependent cardiac parameters (such as those reported in Figure 4), we next compared load-independent parameters between WT and KO_b/c_ mice. Specifically, inferior vena cava (IVC) occlusion was used as a preload reduction maneuver to assess load-independent parameters of contraction and relaxation (the slopes of the end-systolic pressure–volume relationship [ESPVR] and the end-diastolic pressure–volume relationship [EDPVR], respectively).

**Figure 5:**
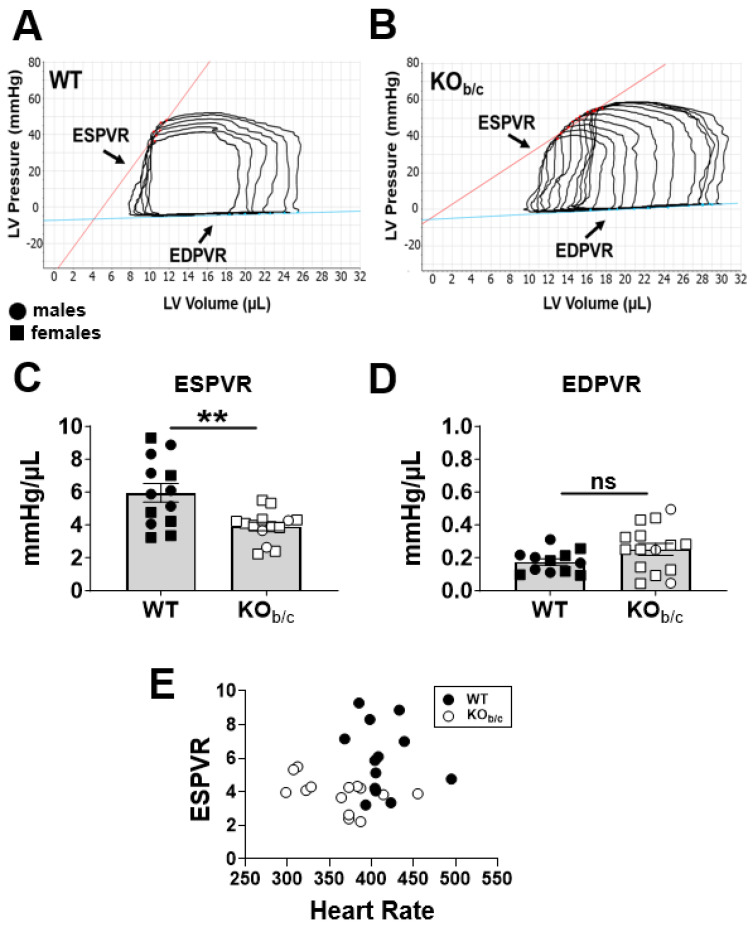
Left intraventricular pressure–volume (PV) assessment during IVC occlusion reveals diminished load-independent contractility in KO_b/c_ mice. Representative PV loops obtained in WT (**A**) and KO_b/c_ (**B**) mice during IVC occlusion used as a preload reduction maneuver to assess load-independent contractility (slope of the end-systolic pressure volume relationship [ESPVR]) and relaxation (slope of the end-diastolic pressure volume relationship [EDPVR]). (**C**) The slope of the ESPVR was significantly reduced in KO_b/c_ mice. (**D**) The slope of the EDPVR was not significantly different between WT and KO_b/c_ mice. (**E**) Plotting ESPVR against heart rate for individual mice illustrates that ESPVR is independent of heart rate, supporting that although KO_b/c_ mice have a slower heart rate than WT during this experiment, this does not account for the observed reduction in their ESPVR. Data presented as mean ± SEM, n = 12–15 per group. Outliers were defined a priori as any point >2 standard deviations from the mean and were excluded from analysis. A single WT outlier was excluded from the EDPVR data set. A single KO_b/c_ outlier was excluded from the ESPVR data set. A significant difference between WT and KO_b/c_ groups is indicated in the figure by ** *p* < 0.01 calculated using Student’s unpaired 2-tailed *T*-test; ns (non-significant).

**Figure 6:**
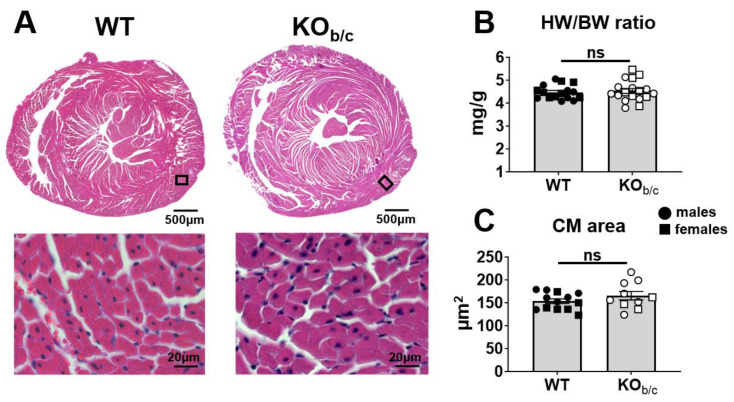
Absence of cardiac hypertrophy in KO_b/c_ hearts. (**A**) Representative low-magnification tiled images, with higher magnified regions of interest (black boxes in low-magnification images), taken of WT and KO_b/c_ heart sections stained with H&E. (**B**) The HW/BW ratio, an index of heart size, was not significantly different between WT and KO_b/c_ mice. (**C**) There was also no significant difference in cross-sectional area between genotypes. Data presented as mean ± SEM, n = 16–18 per group (panel **B**) or 13–10 per group (panel **C**). Outliers were defined a priori as any point >2 standard deviations from the mean and were excluded from analysis. A single WT outlier was excluded from the HW/BW ratio data set. A single KO_b/c_ outlier was excluded from the HW/BW ratio data set. For panel (**B**), the statistical significance was calculated using Student’s unpaired 2-tailed *T*-test. For panel (**C**), the cross-sectional area of 25–29 cardiomyocytes was measured across 5 images taken around the left ventricle and averaged for each individual mouse, with the statistical significance calculated using hierarchal statistical analysis (nested *T*-test). ns (non-significant).

Figure 5A,B illustrate representative PV loops obtained in WT and KO_b/c_ mice during IVC occlusion. Here, we observed a significant reduction in the slope of the ESPVR in KO_b/c_ mice (WT: 6.0 ± 0.6 mmHg/µL, KO_b/c_: 3.9 ± 0.3 mmHg/µL, *p* = 0.003, n = 13 and 14, respectively; Figure 6C), whereas there was no significant difference from WT mice in the slope of the EDPVR (WT: 0.18 ± 0.07 mmHg/µL, KO_b/c_: 0.25 ± 0.04 mmHg/µL, *p* = 0.097, n = 12 and 15, respectively; Figure 5D). Since the animals used to produce the data in Figure 5 were the same as those used to produce the data in Figure 4—and thus the KO_b/c_ mice have a slower heart rate in both data sets—we confirmed that this difference in heart rate did not account for the observed reduction in KO_b/c_ ESPVR by plotting the ESPVR against heart rate for individual mice (Figure 5E). For example, we can see that KO_b/c_ mice with the lowest heart rates (left on the *x*-axis) have some of the highest ESPVR values among the KO_b/c_ group, thus supporting the idea that the reduced ESPVR in KO_b/c_ mice is not attributable to their overall slower heart rate. Overall, the data presented in Figure 5 indicate that KO_b/c_ mice have diminished left ventricular load-independent contractility.

### 2.2. KO_b/c_ Hearts Are Not Hypertrophic at 4–5 Weeks of Age

To assess for hypertrophy in KO_b/c_ mice, we compared WT and KO_b/c_ heart-weight-to-body-weight ratios (HW/BW) and the cross-sectional area of cardiomyocytes within H&E-stained heart sections. Figure 6A shows representative tiled images and higher-magnification regions of interest taken of WT and KO_b/c_ H&E-stained heart sections. There was no significant difference between the HW/BW ratio of WT and KO_b/c_ mice (WT: 4.48 ± 0.07 mg/g, KO_b/c_: 4.53 ± 0.10 mg/g, *p* = 0.680, n = 16 and 18, respectively; Figure 6B). Additionally, we assessed the size of individual cardiomyocytes by measuring the cross-sectional area of 25–29 cardiomyocytes around the left ventricle from individual mice, with the average cardiomyocyte area for each individual mouse shown in Figure 6C. We found no significant difference between WT and KO_b/c_ cardiomyocyte area (WT: 154 ± 6 µm^2^, KO_b/c_: 166 ± 9 µm^2^, *p* = 0.243, n = 13 and 10, respectively). Together, these results suggest that cardiac hypertrophy is not present in 4–5-week-old KO_b/c_ mice.

### 2.3. KO_b/c_ Mice Have No Difference in QT Length but Have Increased QT Length Variation

**Figure 7:**
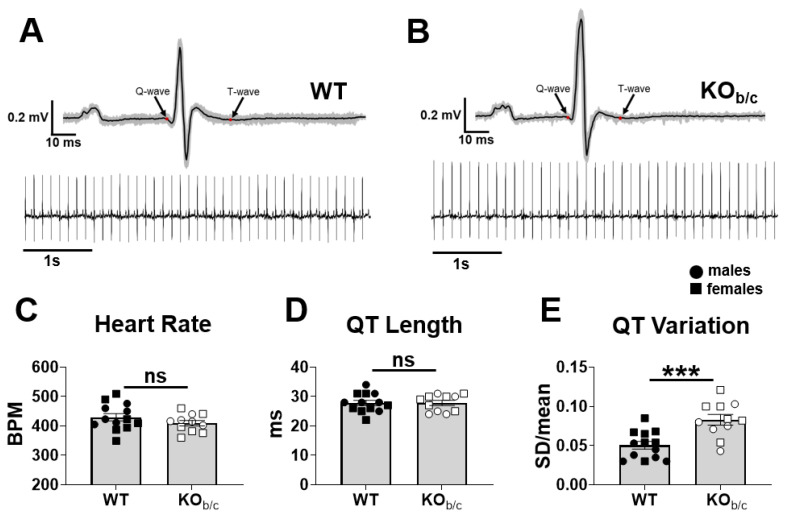
Increased QT length variation in KO_b/c_ mice. Representative average ECG cycles of WT (**A**) and KO_b/c_ (**B**) mice were created from 5 s segments of Lead-I recordings. The black line represents the average trace, with underlying grey lines representing each individual cycle. This method was applied to 30 s Lead-I recordings of WT and KO_b/c_ mice from which QT length and QT length variation were assessed. QT length variation was calculated as the coefficient of variation (SD/mean) across 5 s intervals from a continuous 30 s ECG trace (i.e., 6 × 5 s intervals). (**C**) Heart rates were titrated to between ~350–500 BPM via isoflurane anesthesia. (**D**) There was no significant difference between the length of the QT interval in WT and KO_b/c_ mice. (**E**) The QT length variation in KO_b/c_ was significantly greater than in WT mice. Data presented as mean ± SEM, n = 11–13 per group. A significant difference between WT and KO_b/c_ groups is indicated in the figure by *** *p* < 0.001 calculated using Student’s unpaired 2-tailed *T*-test; ns (non-significant).

To test the hypothesis that the absence of NBCe1-B prolongs the QT interval, 30 s ECGs were recorded in anesthetized WT and KO_b/c_ mice while heart rates were maintained between ~350–500 BPM via titration of isoflurane (WT: 430 ± 13 BPM, KO_b/c_: 410 ± 9 BPM, *p* = 0.228, n = 13 and 11, respectively; Figure 7C). The QT interval was then calculated from an averaged ECG cycle calculated across the 30 s Lead-I recordings for each mouse. As an example, Figure 7A,B illustrate an averaged ECG cycle (black line) across a 5 s segment of Lead-I recordings from WT and KO_b/c_ mice (underlying grey lines represent an overlay of all the individual cycles in the 5 s traces shown below). We found no significant difference in QT interval length between WT and KO_b/c_ mice (WT: 27.8 ± 0.9 ms, KO_b/c_: 27.7 ± 0.8 ms, *p* = 0.937, n = 13 and 11, respectively; Figure 7D). However, we also assessed QT length variation by calculating the coefficient of variation (SD/mean) across 5 s intervals from a continuous 30 s ECG trace (i.e., 6 × 5 s intervals). Using this method, we found that the QT length variation in individual KO_b/c_ mice was significantly greater than that of WT mice (WT: 0.050 ± 0.005, KO_b/c_: 0.083 ± 0.007, *p* < 0.001, n = 13 and 11, respectively; Figure 7E).

### 2.4. Cardiomyocytes from KO_b/c_ Mice Have Diminished Ca^2+^-Transient Amplitude

**Figure 8:**
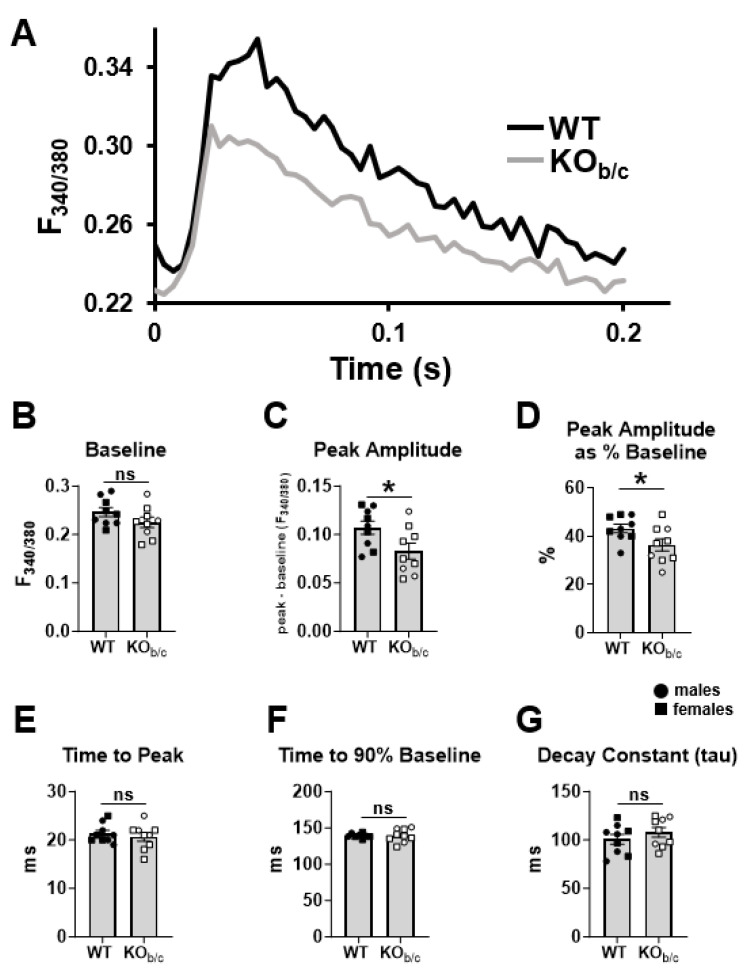
KO_b/c_ cardiomyocytes have reduced Ca^2+^-transient amplitude. (**A**) Representative Ca^2+^ transients recorded in individual cardiomyocytes isolated from WT and KO_b/c_ mice loaded with the intracellular Ca^2+^ indicator Fura-2 AM. Traces represent the average of ~100 consecutive transients recorded in a single cardiomyocyte while paced at 5 Hz. (**B**) There was no significant difference between WT and KO_b/c_ ‘baseline’ F_340/380_ ratio. (**C**) The ‘peak amplitude’ was significantly decreased in KO_b/c_ cardiomyocytes. (**D**) The ‘peak amplitude as % baseline’ (describing the % change from baseline of the Ca^2+^ transient) was also significantly decreased in KO_b/c_ cardiomyocytes. (**E**) There was no significant difference between WT and KO_b/c_ in ‘time to peak’. (**F**) There was no significant difference between WT and KO_b/c_ in ‘time to 90% baseline’. (**G**) There was no significant difference between WT and KO_b/c_ the Ca^2+^ exponential ‘decay constant (tau)’. Data presented as mean ± SEM, n = 8–9 per group with each point representing the mean of 9–12 cells. Outliers were defined a priori as any point >2 standard deviations from the mean and were excluded from analysis. A single WT outlier was excluded from the ‘time to 90% baseline’ data set. A significant difference between WT and KO_b/c_ groups is indicated in the figure by * *p* < 0.05 calculated using hierarchal statistical analysis (nested *T*-test); ns (non-significant).

We tested the effect of congenital NBCe1-B absence on cardiomyocyte Ca^2+^ dynamics by measuring Ca^2+^ transients in freshly isolated cardiomyocytes from WT and KO_b/c_ mice loaded with Fura-2 AM and paced at 5 Hz. Figure 8A shows representative WT (black) and KO_b/c_ (gray) average transients obtained from individual cardiomyocytes. We found no significant difference in ‘baseline’ F_340/380_ between WT and KO_b/c_ cardiomyocytes (WT: 0.246 ± 0.009 F_340/380_, KO_b/c_: 0.225 ± 0.011 F_340/380_, *p* = 0.146, n = 9 for both groups; Figure 8B). However, we found that the F_340/380_ ‘peak amplitude’, which represents the magnitude of Ca^2+^ influx into the sarcoplasm, was significantly decreased in KO_b/c_ cardiomyocytes (WT: 0.107 ± 0.007 F_340/380_, KO_b/c_: 0.083 ± 0.008 F_340/380_, *p* = 0.034, n = 9 for both groups; Figure 8C). Similarly, peak amplitude as a percentage of the baseline (‘peak amplitude as % baseline’), a parameter that reflects the percent change in the Ca^2+^ transient from its baseline, was also significantly reduced in KO_b/c_ cardiomyocytes (WT: 43 ± 2%, KO_b/c_: 36 ± 3%, *p* = 0.040, n = 9 for both groups; Figure 8D). On the other hand, there was no significant difference in the F_340/380_ ‘time to peak’ between WT and KO_b/c_ cardiomyocytes (WT: 21 ± 1 ms, KO_b/c_: 21 ± 1 ms, *p* = 0.516, n = 9 for both groups; Figure 8E), suggesting that the kinetics of Ca^2+^ release are not impaired in KO_b/c_ cardiomyocytes. There was also no significant difference in either ‘time to 90% baseline’ (WT: 139 ± 1 ms, KO_b/c_: 139 ± 3 ms, *p* = 0.960, n = 8 and 9, respectively; Figure 8F) or in the exponential decay constant ‘tau’ (WT: 101 ± 5 ms, KO_b/c_: 108 ± 5 ms, *p* = 0.262, n = 9 for both groups; Figure 8G), both of which reflect Ca^2+^ removal from the sarcoplasm of cardiomyocytes.

## 3. Discussion

The first key finding of this study is that 4–5-week-old KO_b/c_ mice have left ventricular systolic dysfunction with reduced ejection fraction, which to our knowledge is the first demonstration of a mechanical cardiac impairment resulting from the absence of NBCe1-B. Specifically, the echocardiographic and IVC occlusion data (Figure 2 and Figure 5) together suggest that KO_b/c_ mice have a reduced ejection fraction due to a reduction in load-independent contractility. This is likely a result of a diminished systolic phase [Ca^2+^]_i_ peak within individual cardiomyocytes, as we observed a significant reduction in F_340/380_ transient peak amplitude in paced KO_b/c_ cardiomyocytes (Figure 8C,D). That is, since the contractile force generated by a cardiomyocyte is a function of the magnitude of rise in [Ca^2+^]_i_ [35], the reduction in Ca^2+^-transient amplitude within individual KO_b/c_ cardiomyocytes likely underlies the overall decrease in contractility of the KO_b/c_ heart, ultimately resulting in a reduced ejection fraction. Additionally, we found no signs of hypertrophy or impaired relaxation in KO_b/c_ mice, supporting that the impaired contractility is intrinsic to cardiomyocyte function and not a result of pathologic cardiac remodeling.

The second key finding is that while we did not observe QT interval prolongation in KO_b/c_ mice, we did find an increase in QT length variation (Figure 7D,E). This suggests that KO_b/c_ mice are at increased risk for arrhythmia, which is supported by a position statement published in the European Society of Cardiology summarizing evidence that increased QT variation is predictive of arrhythmia and sudden cardiac death [36]. Variation in QT duration at a constant heart rate is a consequence of variability in ventricular repolarization, which itself is affected by variations in the pattern of ventricular activation, conduction velocity, and/or action potential duration (APD) (reviewed in [36]). The latter is the most likely explanation for our findings in the KO_b/c_ mouse, since previous studies have demonstrated a prolonged APD in cardiomyocytes with reduced NBCe1-B expression [32,33]. However, APD is also affected by Ca^2+^, such that a spontaneous release of Ca^2+^ from the sarcoplasmic reticulum during diastole can prolong the APD [37]. Thus, a possible unifying mechanism connecting the absence of NBCe1-B to both systolic dysfunction and increased QT length variation may be the presence of a diastolic Ca^2+^ leak from the sarcoplasmic reticulum.

This possibility of a diastolic Ca^2+^ leak resulting from NBCe1-B loss is further supported by a recent study in which cardiac NBCe1-B expression was partially (~30%) reduced in adult rats using an adenoviral vector [32]. In particular, the authors report no change in stimulated Ca^2+^-transient parameters in isolated cardiomyocytes; however, the frequency of diastolic Ca^2+^ waves were increased in unstimulated cardiomyocytes, a result indicative of spontaneous sarcoplasmic reticulum Ca^2+^ release. Thus, alongside our observations in KO_b/c_ mice of reduced Ca^2+^-transient amplitude in cardiomyocytes and increased QT length variation, these results together suggest that NBCe1-B loss may lead to a diastolic sarcoplasmic reticulum Ca^2+^ leak [38,39,40,41,42,43], a hypothesis that awaits further investigation.

Important differences exist between the previous study of rats with partial reduction in cardiac NBCe1-B and this study of KO_b/c_ mice. Namely, the authors of the previous study provide evidence of cardiac hypertrophy, prolongation of the QT interval, and prolongation of the APD in isolated cardiomyocytes, resulting from the partial reduction in cardiac NBCe1-B [32]. However, no change in cardiac function was found in these rats when assessed by echocardiography [32]. Similarly, in a third study, in which Cre-Lox gene targeting was used to selectively disrupt cardiac NBCe1-B expression in mice (~70% reduction in NBCe1-B mRNA), a significant protective effect of NBCe1-B reduction was found during ischemia–reperfusion injury [44]. However, there was no change in cardiac function found when assessed by intraventricular pressure measurements.

Differences in species, age, as well as complete (this study) versus partial loss (previous studies [32,44]) of NBCe1-B expression may contribute to the differences in study results. Notably, the previous study in rats did not specify sex [32], and the previous study in mice only used males [44]. Important sex differences may exist, as previous studies demonstrate that cardiac NBCe1 activity is diminished in female rats after ovariectomy [45], suggesting that the loss of NBCe1 may play a larger role in female cardiomyocytes. Thus, it is possible that cardiac NBCe1 loss may be more detrimental to females than males. Our data were not sufficiently robust to determine whether sex was an influential factor for our observations, and thus, it remains possible that the features of the group are not reflective of the features of both sexes. Regarding the compensatory upregulation of NBCn1 and NHE1 observed by others in NBCe1 knockdown models, we did not discern a significant difference in their abundance between WT and KO_b/c_ hearts using quantitative PCR (NBCn1: 2^ΔΔCt^ = 0.98, *p* = 0.65; NHE1: 2^ΔΔCt^ = 1.03, *p* = 0.74; n = 3 females,) but cannot discount the possibility that these transporters are post-transcriptionally activated in our model. We note that, according to RNAseq (GEO repository accession GSE223324), ~1500 genes are significantly differentially expressed between WT and KO_b/c_ hearts. Thus, the mechanisms underlying the observed phenotype are likely to be complex.

It is important to note that we were restricted to using animals between 4–5 weeks of age since the early mortality in KO_b/c_ mice (50% mortality at 5 weeks) made it impractical to use adult KO_b/c_ mice [29]. Therefore, it is possible that we observed KO_b/c_ mice at an early stage of heart failure, where there is adequate compensation such that structural changes have yet to develop. Furthermore, the small size of the mice necessitated an invasive approach to intraventricular catheterization, which restricted our ability to titrate the heart rates of WT and KO_b/c_ mice like we did for the less invasive echocardiographic and ECG assessments. Specifically, for the intraventricular catheterization experiments, anesthetic depth was determined to be reached once the pedal reflex was absent. This level of anesthesia apparently had a greater effect on this particular cohort of KO_b/c_ mice (although this was not the case in general), as we observed a significantly slower heart rate in KO_b/c_ mice during these intraventricular catheterization experiments. Intriguingly, however, despite the slower heart rate, we observed no significant difference between WT and KO_b/c_ volume-dependent parameters (Figure 4). Moreover, KO_b/c_ stroke volume was maintained (Figure 2E) at the expense of a greater EDV (Figure 2D), which all together suggests that the Frank–Starling mechanism is adequately compensating for the innate impairment in myocardial contractility.

Thus, we believe that the intrinsic reduction in KO_b/c_ cardiac contractility, due to a decreased cardiomyocyte Ca^2+^-transient amplitude, requires an elevated EDV to maintain adequate end-diastolic pressure, stroke volume, and overall cardiac output. We cannot rule out the possibility that, if KO_b/c_ mice could grow older, this compensation may start to fail, leading to greater elevations in end-diastolic volume and pressure that could lead to structural changes such as concentric and eccentric hypertrophic remodeling. However, the absence of hypertrophy (or to be clear, the absence of detectable signs of hypertrophy within the short lifespan of these mice), is unexpected only inasmuch as it is different from observation of the virally induced NBCe1-knockdown rat model [32]. As an observation in itself, it is not unusual that left ventricular dysfunction (or even heart failure) can develop without hypertrophy when the pathology is unrelated to pressure and/or volume overload.

The literature relating NBCe1 to cardiac hypertrophy is complicated. Some studies correlate pressure-induced hypertrophy with increased NBCe1 expression (e.g., [46]). Cardiac hypertrophy has not been reported in humans with NBCe1 loss. In that context, it is perhaps the observation of hypertrophy in the NBCe1-knockdown rat model, without pressure overload, which is unexpected. In that model, 28 days of virally induced NBCe1 knockdown in adult rats is consistent with an increase in frequency of spontaneous Ca^2+^ release from the SR (without a change in peak amplitude) and an increase in RNA for NBCn1 and NHE1 that could compromise NCX activity and cause a hypertrophic elevation in intracellular Ca^2+^ [32]. Those authors are careful to note that this is just a hypothesis and that the actual cause may be far more complex. In contrast, our mice lack NBCe1-B congenitally and barely survive 28 days. We report a reduced peak amplitude of Ca^2+^ release and no change in the abundance of RNA for NBCn1 or NHE1. Thus, our mice do not clearly conform to the rat model/hypothesis regarding hypertrophy. The reasons for the disparity are unknown. Evidently, these two models of NBCe1 ablation have very different cellular effects but ought not be taken as evidence that KO_b/c_ mice are resistant to hypertrophy.

In summary, we have described a phenotype of left ventricular systolic dysfunction in KO_b/c_ mice that is likely a result of diminished Ca^2+^-transient amplitude in cardiomyocytes. Additionally, the congenital absence of NBCe1-B led to increased QT length variation. Overall, this study adds to the growing body of literature in rodent models supporting the notion that alterations in acid-base transporter expression/activity can be central drivers of cardiac pathophysiology. However, it is critical to take view these observations in context: these data report the cardiac phenotype of KO_b/c_ mice rather than the effects of NBCe1-B loss from the heart. This is a crucial distinction because NBCe1-B and NBCe1-C are expressed in locations throughout the body including the central and peripheral nervous systems as well as various endocrine glands and throughout the gastrointestinal system. To assess the cardiac-specific role of NBCe1-B, confirmatory studies will need to be performed in mice with a cardiomyocyte-specific deletion of NBCe1-B. It is only in a cardiomyocyte-specific deletion model that mechanistic studies elucidating the role of NBCe1-B in the heart can be performed with full confidence that the data will not influenced by unmeasured neuronal, endocrine, or nutritional disturbances. Our data suggest that the cardiac function of individuals with NBCe1 loss ought to be carefully monitored and reported in future, both to ensure that development of heart failure is not missed and to assess the translational usefulness of our model.

## 4. Materials and Methods

### 4.1. Mice

The generation and genotyping of the KO_b/c_ mouse on a C57BL/6J background have been previously reported [29], with mice genotyped at 2 weeks of age. For this study, heterozygous parents were produced by backcrossing heterozygous mice with verified wild-type (WT) C57BL/6J mice (Jackson Laboratory, Bar Harbor, ME, USA). Heterozygous progeny (F6 to F17 generation, making them at least 99% genetically identical) were crossed to produce experimental WT and KO_b/c_ mice for this study. As previously reported, KO_b/c_ mice exhibit increased mortality (50% at 5 weeks [29]) so we were constrained to working with mice between 4–5 weeks of age in order to maximize the likelihood of survival during study. The total number of WT mice used was 38 and the total number of KO_b/c_ mice used was 37.

### 4.2. Blood Pressure

Non-invasive blood pressure measurements were taken using the CODA monitor tail-cuff system (Kent Scientific, Torrington, CT, USA). The mice were placed in a restraint that allows free access to the tail and placed on a heating pad (37 °C). The mice were left for 10 min to thermoregulate and acclimatize to the restraint before their blood pressure was taken.

### 4.3. Echocardiography

The mice were anesthetized with 1% isoflurane, and their heart rate was maintained between 300–500 BPM (beats per minute) with 0.5–1% isoflurane. The mice were kept on a warming pad (37 °C), secured in a supine position, and their chest hair was removed using calcium hydroxide cream (Nair). A 14 MHz probe (GE Vivid E9) was used to image the left ventricle in the short axis. Two-dimensional M-mode recordings of the left ventricle were obtained at the papillary muscle level and used for analysis of cardiac function. The width of the left ventricular anterior and posterior walls (LVAW and LVPW) and the left ventricular internal diameter (LVID) during systole (s) and diastole (d) were manually measured from the M-mode images. Fractional shortening (FS) was calculated as FS = [(LVIDd − LVIDs)/LVIDd] × 100%. End-systolic volume (ESV) and end-diastolic volume (EDV) were calculated from the LVIDs and LVIDd using the method established by Teichholz et al. [47,48]: ESV = 7/[2.4 + LVIDs] × [LVIDs]^3^ and EDV = 7/[2.4 + LVIDd] × [LVIDd]^3^. Stroke volume (SV) was calculated as SV = EDV − ESV. Ejection fraction was calculated as EF = SV/EDV × 100%.

### 4.4. Electrocardiogram (ECG) Recording and Analysis

The mice were anesthetized with 1% isoflurane, and their heart rate was maintained between 300–500 BPM with 0.5–1% isoflurane. The mice were kept on a warming pad and secured in a supine position on a Mouse Monitor S (Indus Instruments, Webster, TX, USA) and recorded for 30 s according to the recommended manufacturer’s settings. Voltage and time parameters from Lead I were exported to MATLAB R2020b (version 9.9.0) and analyzed using the ‘Signal Analyzer App’ from the Signal Processing Toolbox (available through MATLAB Add-On Explorer) and BioSigKit [49] (available at https://github.com/hooman650/BioSigKit; accessed on 22 March 2019) modified in-house to calculate QT intervals (code available upon request). In particular, the T-wave was identified using a modification of the method demonstrated by Zhang et al. [50]; here, the T-wave was defined as the point after the J-wave where the derivative of the average trace equals zero. This enabled reliable identification of entirely positive T-waves (i.e., those that do not return to the isoelectric baseline) as well as T-waves with a negative undershoot. QT length variation was calculated as the coefficient of variation (SD/mean) across 5 s intervals from a continuous 30 s ECG trace (i.e., 6 × 5 s intervals) from Lead I.

### 4.5. Intraventricular Admittance Catheter-Derived Pressure–Volume (PV) Analysis

The mice were anesthetized using 1% isoflurane and were maintained at a surgical plane of anesthesia (1–2% isoflurane) as assessed by the absence of pedal reflex (toe pinch). The mice were kept on warming pad (37 °C), secured in a supine position, and their chest and abdominal hair was removed using calcium hydroxide cream (Nair). The trachea was intubated using a 22 g polyethylene catheter and ventilated with room air supplemented with O_2_ (1 L/min) at a rate of 150–200 strokes/min and tidal volume of 200–500 µL using a small animal ventilator (Inspira ASV, Harvard apparatus, Holliston, MA, USA). The chest cavity was accessed by making a horizontal surgical incision below the xyphoid process, and the diaphragm was bluntly dissected to expose the heart apex. Cautery was used to minimize bleeding. A 27 g needle was used to puncture the apex of the myocardium to advance a 1.2 F microtip PV catheter (Transonic Systems, Ithaca, NY, USA) into the left ventricular cavity. PV signals were continuously recorded using an ADV500 PC conductance system (Transonic Systems) coupled to a PowerLab/8SP analog-to-digital converter (AD Instruments, Colorado Springs, CO, USA). PV loop data were analyzed using LabChart (v8, AD Instruments). To obtain load-independent measures of contractility, preload reduction was accomplished by threading silk suture around the inferior vena cava (IVC) and gently pulling up for ~5 s to briefly reduce blood flow to the right atrium (i.e., IVC occlusion). The end-systolic and end-diastolic PV relationships were assessed by fitting a linear equation to end-systolic/diastolic PV points of 5–16 cardiac cycles. Additional points from cardiac cycles that resulted in a negative slope were attributed to volume/pressure reduction impairing cardiac perfusion and were excluded.

### 4.6. Histological Analysis

The mice were euthanized by isoflurane (5%) inhalation overdose followed by cervical dislocation. Their hearts were excised and placed in a 1 M KCl solution (prepared in PBS: 46-013-CM, Corning Life Sciences, Corning, NY, USA) to arrest the heart in diastole. The hearts were sectioned into 500 µm transverse sections through the left ventricle using a metal scaffold to ensure the slices occurred in similar locations between hearts. The tissue was immediately placed in 4% paraformaldehyde for 24 h at 4 °C and then stored in a 70% ethanol solution at 4 °C until embedding. The tissue was then embedded in paraffin blocks using standard embedding procedures. Briefly, the tissue was dehydrated through incubations in 80% and 95% ethanol, 45 min each, followed by 3 changes of 100% ethanol, 1 h each. The tissue was cleared through 2 changes of xylene, 1 h each, and placed in molten paraffin overnight (H-PF, General Data, Cincinnati, OH, USA), followed by embedding in paraffin blocks. The gross heart sections were further sectioned at a thickness of 5 μm, mounted on frosted slides, and dried at 37 °C overnight. For hematoxylin and eosin staining (H&E), sections were deparaffinized, rinsed with DI water, and stained with H&E according to manufacturer’s instructions (Abcam, Waltham, MA, USA; hematoxylin ab220365, eosin ab246824). Sections were dehydrated with graded ethanol solutions and xylene and mounted for light microscopy. High-magnification (40×; Leica DM 6B upright microscope; Leica Microsystems, Deerfield, IL, USA.) images were taken around the left ventricle (5 images per section), and the cross-sectional area of 25–29 cardiomyocytes per mouse, in which the nucleus could clearly be visualized in the center of the cell, was measured using FIJI software (ImageJ; https://fiji.sc/; accessed 27 August 2019).

### 4.7. Cardiomyocyte Isolation

Cardiomyocytes were isolated from fresh heart tissue using a Langendorff-free method based on the methods described by Ackers et al. [51] and Farrugia et al. [52]. Briefly, mice were euthanized by an intraperitoneal injection of Fatal-Plus (sodium pentobarbital, 0.3 mg/g) and after reaching proper anesthetic depth (assessed by absence of pedal reflex) the thoracic cavity was opened. The descending aorta and inferior vena cava were cut, and the right ventricle was rapidly perfused with 10 mL of ice-cold EDTA buffer solution over ~3 min (in mM: 130 NaCl, 5 KCl, 0.5 NaH_2_PO_4_, 10 HEPES, 10 Glucose, 10 BDM, 10 Taurine, 5 EDTA). The ascending aorta was then clamped, and the heart was excised to a dish containing warm (37 °C) EDTA buffer, and 15 mL of pre-warmed EDTA buffer was injected into the left ventricle. The heart was then transferred to a Petri dish containing pre-warmed (37 °C) collagenase buffer (enzymes [in mg/mL: 0.5 Collagenase II, 0.5 Collagenase IV, 0.05 Protease XIV; Worthington Biochemical] prepared in magnesium buffer [in mM: 130 NaCl, 5 KCl, 0.5 NaH_2_PO_4_, 10 HEPES, 10 Glucose, 10 BDM, 10 Taurine, 1 MgCl_2_]), and the left ventricle was perfused with 25–40 mL of pre-warmed collagenase buffer until digested. The left ventricle was separated and moved to a new dish of pre-warmed collagenase buffer and teased apart to release individual cardiomyocytes. The cell suspension was then incubated in 5 mL of collagenase buffer for 5 min at 37 °C, gently mixed, and incubated for another 5 min before filtering with 100 µm mesh into 5 mL of 5% fetal bovine solution (FBS, Thermo Fisher Scientific, Grand Island, NY, USA; prepared in magnesium buffer) in order to stop the digestion. The cell suspension was then centrifuged at 51 G for 2 min to loosely pellet the cells and then resuspended in 10 mL of fresh 5% FBS. Reintroduction of Ca^2+^ took place over 4 changes of supernatant each with a 10 min incubation (pelleting cells in between each change), increasing the [Ca^2+^] with each change (in mM: 0.07, 0.27, 0.68, 1.35) resulting in a final [Ca^2+^] of 1.35 mM. The Ca^2+^ reintroduction solutions were prepared by diluting Ca^2+^-containing HCO_3_^−^ buffer (in mM: 118 NaCl, 5 KCl, 10 Glucose, 5 HEPES, 20 NaHCO_3_^−^, 0.8 MgCl_2_, 1.35 CaCl_2_, 1.2 MgSO_4_; aerated with 5% CO_2_, 21% O_2_, and 74% N_2_, pH 7.4) with magnesium buffer to obtain the correct [Ca^2+^].

### 4.8. Ca^2+^ Transient Recordings and Analysis

A total of 5 µL of Pluronic F-127 (20% *w/v* prepared in DMSO; ThermoFisher, P6867) and 1 µL Fura-2 AM (1 mM prepared in DMSO; Abcam, ab120873) were added to a 1 mL aliquot of the cell suspension, leading to final concentrations of 0.1% and 1 µM, respectively, and incubated for 20 min protected from light. The cells were then loaded into a perfusion chamber (IonOptix, Westwood, MA, USA) and perfused with the Ca^2+^-containing HCO_3_^−^ buffer at 3 mL/min maintained at 30 °C. The cells were perfused for 10 min to allow for desertification of the Fura-2 AM before the start of the experiment. The cardiomyocytes were stimulated using platinum wires positioned on opposite sides of the chamber with a 4 ms positive waveform at a frequency of 5 Hz using the MyoPacer stimulator (IonOptix). The voltage was adjusted until ~50% of the cell field was contracting, generally between 10–20 V. Evoked Ca^2+^ transients were recorded using a dual-excitation spectrophotometer (IonOptix). Specifically, the 510 nm emission at alternating excitation wavelengths of 340 nm and 380 nm was recorded in individual cardiomyocytes for ~60 s followed by ~20 s focused on an empty field of view to record the background. The recording sampling frequency was set to 250 Hz. The background was subtracted from the 340 nm and 380 nm emission recordings, and the resulting 340/380 ratio (F_340/380_) waveform was analyzed using IonWizard software (IonOptix, version 7.3.0). For each cell, the F_340/380_ waveform was averaged into a single transient for analysis (~100 individual transients).

### 4.9. Real-Time PCR

Hearts from female WT and KO_b/c_ mice (n = 3 per group) were excised and stored in RNAlater (Thermo Fisher Scientific) until homogenization and RNA purification using the RNeasy Micro Kit (Qiagen, Germantown, MD, USA). Resulting total-RNA was quantified using a Nanodrop 2000 (ThermoFisher). Real-time PCR was performed according to the manufacturers’ recommendations using PrimePCR SYBR green assays (BioRad, Hercules, CA, USA) for mouse NBCn1 (*Slc4a7*: qMmuCID0021763), NHE1 (*Slc9a1*: qMmuCID0010387) and the control genes *Gapdh* (assay qMmuCED0027497) and *Pol2ra* (assay qMmuCID0005230). NBCn1 and NHE1 transcript abundance was normalized for the combined average abundance of Gapdh and Pol2ra [53] using the Livak equation.4.10. Statistical Analysis

The results are presented as mean ± SEM, with *n* referring to the number of animals studied. After exploratory data collection to n = 6 for each experiment, the ultimate sample size required for 80% power and a significance threshold of α = 0.05 for the difference between means and their common standard deviation was guided using an online calculator hosted by the University of British Columbia (StatSpace, https://www.stat.ubc.ca/~rollin/stats/ssize/n2.html, accessed 1 September 2019). Outliers for any individual parameter were defined a priori as any point >2 standard deviations from the mean and were excluded from analysis (amounting to no more than 2 data points excluded per group). The number (*n*) of outliers for any given parameter is reported in the appropriate figure legend. In all the analyses, the threshold of *p* < 0.05 was used to determine statistical significance. Normality of the data was tested in GraphPad (v9.0) using the D’Agostino–Pearson omnibus test. Statistical significance between WT and KO_b/c_ groups was calculated using Student’s *T*-test (2-tailed). For cell-based studies (Figure 6C and Figure 8), we performed hierarchical statistical analyses (nested *T*-tests) to account for inter-subject variability and dependence between samples. Due to the low fertility and high mortality of KO_b/c_ mice, we were obliged to use male and female mice non-discriminately throughout this study based on availability at the time of each experiment. As a consequence, we find that our data are not sufficiently robust to assess the importance of sex as a biological variable [54]. All the analyses were performed in Microsoft Excel, Prism GraphPad version 9, G × Power (http://www.gpower.hhu.de, accessed 25 July 2024), or IBM SPSS (v29). The figures were prepared using Microsoft PowerPoint v2408, Microsoft Excel v2408, Prism GraphPad version 9, and BioRender.com.

## Data Availability

The original contributions presented in this study are included in this article, further inquiries can be directed to the corresponding authors.
